# Recent Advances in Printed Chipless Passive Inductively Coupled LC-Based Telemetric Systems for Smart Products: A Scoping Review

**DOI:** 10.3390/s26103233

**Published:** 2026-05-20

**Authors:** Edoardo Cantù, Nicola Francesco Lopomo, Claudio Pirola, Emilio Sardini

**Affiliations:** 1 INXENSE s.r.l., 25123 Brescia, Italy; edoardo.cantu@inxense.it; 2 Department of Design, Politecnico di Milano, 20133 Milan, Italy; nicola.lopomo@polimi.it; 3 Department of Information Engineering, University of Brescia, 25123 Brescia, Italy; emilio.sardini@unibs.it

**Keywords:** telemetric systems, passive system, inductive coupling, LC-based system, printed electronics, inkjet printing (IJP), aerosol jet printing (AJP), screen printing (SP), smart products, printed telemetric systems

## Abstract

Telemetric systems are particularly valuable in applications where remote data acquisition and automatic transmission allow effective monitoring of local characteristics. Among the different telemetric approaches, passive wireless systems based on inductive coupling are particularly attractive because they enable sensor interrogation without onboard power storage. Printed electronics (PE) offer several advantages in the realization of such systems, including a wide selection of functional materials, reduced production costs, possibility of rapid prototyping and complete customization. This allows for the development of smart products by embedding sensors and electronics directly into existing objects without significantly altering their geometry or weight. In light of this, the aim of this scoping review is to explore key factors in implementing chipless passive inductively coupled LC telemetric systems via PE. Given the growing interest in smart products, this scoping review serves as a starting point for the design and implementation of smart products specifically on printed passive inductively coupled LC telemetric systems, addressing their development. To better understand the identified solutions, we first outlined the requirements and characteristics of ideal chipless passive LC-based inductively coupled telemetric systems. Then, we provided a comprehensive analysis of conductive materials and substrates, manufacturing technologies, and the design and performance of printed inductors and associated readout architectures.

## 1. Introduction

Telemetric systems are particularly valuable in applications where remote data acquisition and automatic transmission allow effective monitoring of local characteristics [1]. Among the different telemetric approaches, passive wireless systems based on inductive coupling are particularly attractive because they represent an ideal solution for applications where wired sensors and onboard power sources are impractical and not feasible [2].

These characteristics are especially valuable in diverse fields, ranging from environmental and mechanical monitoring (e.g., strain or pressure) to healthcare and biomedical sectors, including wearable devices [3,4].

Chipless passive inductively coupled LC-based telemetric systems typically consists of an in situ sensor and a transmitting element, designed to detect a single physical/chemical quantity. More recently, these systems have advanced to allow for the simultaneous measurement of multiple parameters [5]. This capability is crucial for effective monitoring, error compensation, and precise analysis of coupled physical/chemical phenomena, as well as for correcting environmental drifts such as temperature effects [6,7]. The integration of several sensing functions into a unified system significantly streamlines both biomedical and industrial applications. In fact, this approach achieves superior miniaturization and efficiency, leading to a substantial reduction in both hardware complexity and the intrusiveness of the system [3,7].

From a technical standpoint, multi-parametric passive LC-based telemetric systems can be realized by combining multiple sensing components with the transmitting ones [5]. While this feature makes these systems highly attractive, on the other hand, a primary obstacle in advancing multi-parametric passive LC-based telemetry is the interference caused by inductive coupling (i.e., cross-talk) when multiple transmitting elements are integrated within a compact space [8]. To overcome this issue, several techniques have been proposed in the literature. One prominent approach involved the use of a specific stacked antenna geometry [9]. Experimental studies verified that the proposed configuration was able to effectively suppress mutual inductance, thus allowing dense sensor integration. Further alternative strategies included the use of branching inductors [10] or symmetric circuit designs [7]. This latter technique [7] proved to be particularly promising since it allowed the simultaneous monitoring of three different parameters using only two capacitive sensors. In this configuration, two parameters were specifically derived from two independent resonance frequencies, whereas the third one was derived from the quality factor (Q-factor), which varied according to the parasitic resistances of the inductors. Furthermore, partially overlapped antenna configurations [5,11] were successfully employed to minimize the coupling coefficient and thus obtain accurate identification of the distinct resonance frequencies for each sensor.

Further information to consider when designing and developing telemetric systems is related to the definition of the manufacturing process and materials [12]. In general, they have been traditionally developed using transmitting elements produced using printed circuit boards (PCBs) [13,14]. In the last few decades, printed electronics (PE) technologies have also been introduced and exploited in this context. In fact, the use of PE offers advantages over traditional manufacturing methods, including lower production costs and compatibility with a wide range of functional materials—such as conductive inks—and various substrates, including flexible materials such as natural cellulose fibres (e.g., common paper) or polyimide (e.g., Kapton). Indeed, the latter approaches have allowed the development and exploitation of conformable solutions. Furthermore, PE technologies allow for rapid prototyping of the sensing components, by either directly printing on objects [15] or functionalizing substrates suitable for integration [16]. These capabilities enable the development of smart products by embedding sensors and electronics directly into existing objects. Therefore, unlike traditional systems based on rigid substrates, such as PCBs, PE technologies allow for easier integration of electronics without significantly altering the shape, volume, or weight of the original products. Among the different solutions identified in the scientific literature, inkjet printing (IJP) [17], screen printing (SP) [18] and aerosol jet printing (AJP) [19] are the most prevalent technologies used for the fabrication of chipless passive inductively coupled telemetric systems. Indeed, PE facilitates the embedding of sensing components, thanks to its minimal required spatial footprint and its versatile positioning. Given these advantages of using PE technologies in realizing passive LC-based telemetric systems, a deep understanding of their design and production processes is mandatory, so as to better exploit their overall potential.

Within this context, it is worth noting that the growing body of literature on printed electronics, passive wireless sensing, and smart product integration has been partially addressed by previous reviews, which can be mainly classified into three overlapping categories according to their specific scope. The first group of reviews addressed printed electronics broadly, covering conductive inks, substrates, and fabrication technologies—including inkjet, screen, aerosol jet, and gravure printing—applied to a wide range of electronic components and devices [20]. While these works are indeed valuable since they provide overviews of materials and manufacturing approaches, they do not focus on the specific architecture of chipless passive inductively coupled LC-based systems or address the design chain from printed inductors to wireless readout in the context of embedding smart products. The second group of reviews focused specifically on passive wireless LC resonant sensors, analysing their operating principles, equivalent circuit models, readout strategies and applications in domains such as biomedical monitoring and harsh-environment sensing [21,22,23,24]. These works are architecturally aligned with the possibility of having passive LC-based telemetry; however, they do not specifically address the realization of such systems via printed electronics, and therefore they do not discuss the constraints, opportunities, and material/process trade-offs that arise when the inductor, capacitor, and sensing element are all fabricated through additive printing on flexible or unconventional substrates. The third group of reviews focused on printed or additively manufactured wireless sensors, including batteryless RF resonators and NFC/RFID-based flexible tags [25,26]. While these works intersect with the possibility of implementing wireless telemetric solutions, they either cover a broader range of wireless sensing modalities beyond purely passive inductive coupling, include chip-based active components that fall outside the battery-free passive LC paradigm, or they do not specifically frame their analysis around smart product integration and the systematic evaluation of the full system—from conductive material selection and substrate compatibility to inductor/capacitor design and readout performance.

To the best of the authors’ knowledge, no existing review simultaneously addresses all these dimensions, including: (1) chipless passive inductively coupled LC-based telemetric architectures, (2) realized exclusively through printed electronics fabrication processes, and (3) oriented toward integration in smart products with a minimal component count. The present work is positioned at this intersection, with the aim of providing specifically a consolidated reference for researchers and engineers approaching the co-design of printed passive inductively coupled LC-based telemetric systems.

Therefore, our goal was to explore within this very specific context all the available materials—including both substrates and functional inks—along with appropriate designs and manufacturing processes. Within this scope, Section 2 underlines the theoretical framework concerning the requirements and characteristics of this kind of telemetric system. Section 3 describes the method used to carry out the scoping review and further includes a description of the common approaches and measurement techniques found in the literature before finally describing the reviewed works in terms of geometry, manufacturing technologies, materials, performance, and applications. While the paper selection process was documented using PRISMA guidelines to provide a transparent, reproducible and systematic search framework, the overarching aim of the review was to map the existing literature and identify technological gaps regarding printed passive LC systems. Section 4 analyses and discusses in detail the characteristics of the reviewed systems, with a specific focus on the equivalent antenna model, manufacturing criticalities, and overall measuring performance.

## 2. Requirements and Characteristics of Ideal Chipless Passive LC-Based Telemetric Systems

**Figure 1:**
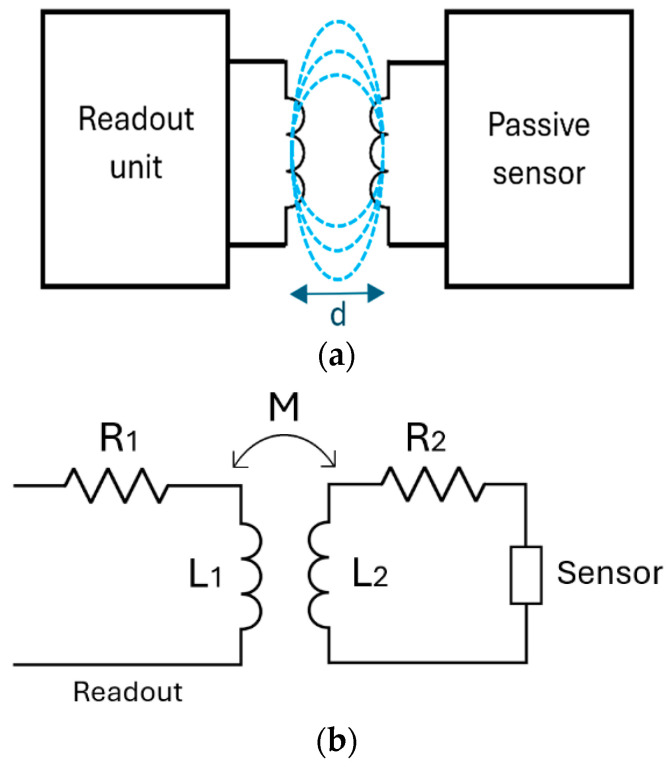
Representation of an ideal telemetric system (**a**) and equivalent circuit of the system (**b**).

Chipless passive inductively coupled LC telemetric systems consist of a passive sensing stage and a receiving element including front-end electronics (i.e., the readout unit), which is magnetically coupled via a mutual inductor. As shown in Figure 1a, the primary coil of the mutual inductor is linked to the readout unit, while the secondary coil is connected to the sensing component. The readout unit deploys two different tasks; specifically, thanks to the inductive coupling: (1) it powers up the passive sensor, thus ensuring the measuring tasks, and (2) it reads the measurements provided by the passive sensor. An ideal passive LC-based telemetric system should be immune to changes in the distance *d* or orientation between the transmitting and the receiving components, thus ensuring reliable monitoring of the target physical/chemical quantities. Moreover, an ideal passive LC-based telemetric system should also have high resolution and sensitivity.

**Figure 2:**
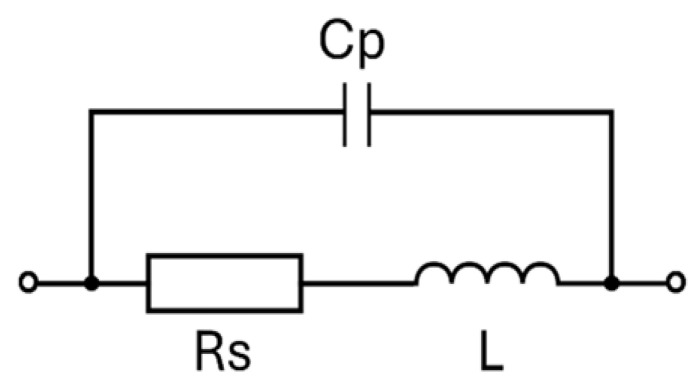
Equivalent model of a real inductor.

Given these characteristics, it is necessary to consider the equivalent circuit of the system—as represented in Figure 1b. In particular, it is worth considering the coupling coefficient *M* of the mutual inductor. The equivalent circuit is composed of the sensor and the equivalent stage. *L*_1_ and *L*_2_ represent, respectively, the primary and the secondary coil of the mutual inductor, whereas *R*_1_ and *R*_2_ represent the equivalent resistances. Ideally, a passive LC-based telemetric system must have a high value of sensitivity, which requires it to have a high quality factor (*Q*) for the secondary inductor, ideally tending towards infinity. The most common approach to represent a real system is through an equivalent circuit consisting of an inductor (*L*) and a resistor (*R_s_*) in series, all in parallel with a capacitor (*C_p_*), as shown in Figure 2. *R_s_* and *C_p_* are parasitic elements representing, respectively, the resistance of the conductive path and the capacitance between the inductor windings [27].

**Figure 3:**
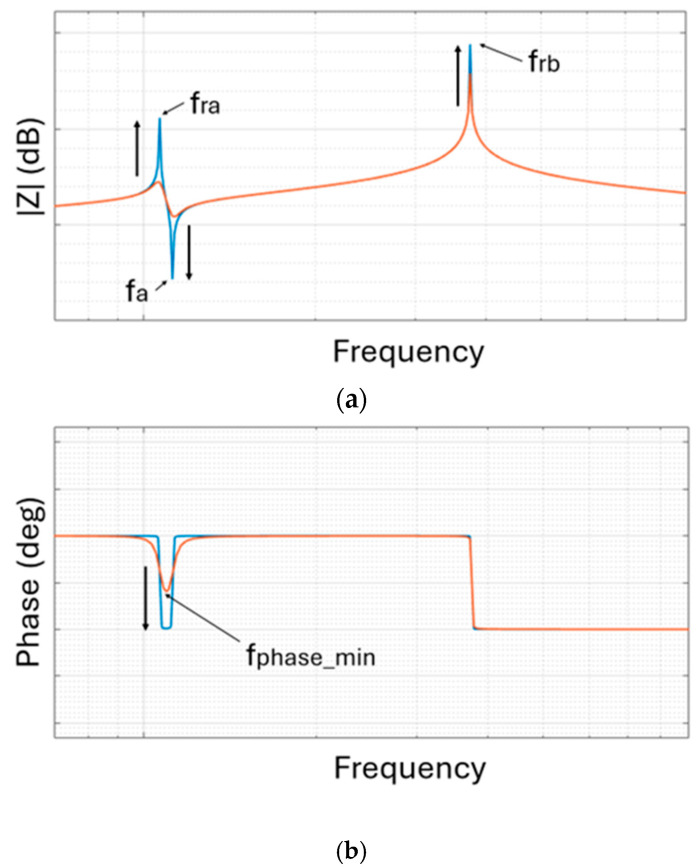
Modulus (**a**) and phase (**b**) behaviour of the telemetric system for increasing the *Q* value of the secondary inductor. The blue lines represent behaviour with a higher quality factor; the orange lines represent behaviour with no increase in quality factor.

Equivalent models of the inductor composed only of resistance and inductance in series, as well as more complex models, can be found in the scientific literature [28]. For example, an application-specific and complex equivalent model took into account skin and proximity effects, parasitic capacitance of the spiral windings to the ground and substrate loss [29]. Considering the equivalent model in Figure 2, the need to have the highest possible value of *Q* means that the inductor must have the lowest resistance *R_s_*. The highest possible value of *Q* is then required to obtain a behaviour of the secondary inductor at the operating frequency that is equivalent to an ideal inductor. In addition, a high value of *Q* also influences the behaviour of the system in frequency. As shown in Figure 3, the system impedance modulus and phase seen from the readout unit present several variations. The peaks at the resonance frequencies (*f_ra_* and *f_rb_*), at the minimal phase frequency (*f_phase_min_*) in proximity to *f_ra_* and *f_rb_*, and at the anti-resonant frequency (*f_a_*) increase as *Q* increases.

A significant challenge in reviewing this specific domain of telemetry is the overlapping nomenclature utilized across different research communities. Devices operating via inductive coupling are frequently referred to interchangeably as passive LC sensors, inductively coupled telemetric systems, and—particularly in the context of IoT applications—near-field chipless RFIDs.

For the clarity of this scoping review, our clearly defined review object is printed passive LC-based telemetric systems. Furthermore, because the overall performance, quality factor *Q*, and reading range of such systems are fundamentally dictated by the characteristics of the inductive coupling, the design, conductive materials, and fabrication techniques of the printed inductors represent the primary technological bottleneck. Consequently, while this review touches upon whole-system architectures and readout units, the core contribution of this work is a dedicated mapping of the state of the art in the fabrication and optimization of printed inductive components.

## 3. Methods

**Figure 4:**
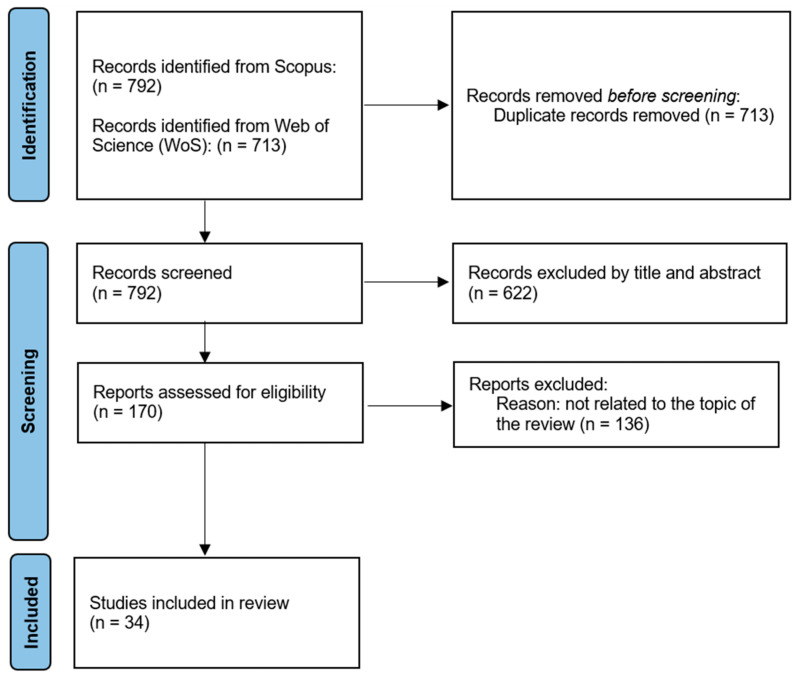
PRISMA flow diagram for the systematic review.

The scoping review was carried out to deepen the design and implementation of printed inductively coupled LC telemetric systems, with particular attention to the production/manufacturing processes used to realize the inductors. The focus of the research was on printing techniques, employed materials (conductive and insulating inks/pastes and substrates) and geometrical layouts. Furthermore, the different fields of application were also mapped to provide useful insights with respect to the environments and possible constraints. Specific attention was also paid to the overall performance of the analysed inductors, defined in terms of resistance, inductance and quality factor values. The proposed results were obtained by systematically searching the literature in 2 online databases (i.e., Scopus and Web of Science), because the objective was to map, categorize, and analyse a heterogeneous body of literature rather than answer a narrowly defined question through formal quantitative or comparative synthesis. Studies were considered eligible if they reported printed or printing-enabled chipless passive inductively coupled sensing or telemetric systems, or enabling printed components directly relevant to such systems, including resonant structures and readout-related elements, such as antennas. Studies focused exclusively on unrelated printed electronics, conventional printed circuit boards, or inductive components without sensing or telemetric relevance were excluded during screening. The time window was limited to the recent literature (2018–2025) in order to capture current technological developments, while earlier seminal and review articles identified through citation tracking were used to contextualize the field. For the sake of transparency and clearness and to support a systematic approach to the literature search, Figure 4 shows the PRISMA flow diagram. All the numbers of results obtained from searches are updated to 30 October 2025.

Before starting to conduct the systematic approach to support the scoping review, a preliminary search was initially performed aiming to optimize the framing of the research questions we wanted to answer. A wide set of papers focused on printed inductive, contactless or battery-free sensors were analysed so as to understand if passive inductors realized via printed electronics were useful for designing telemetric systems. This search produced 17 valid results on Scopus and 15 valid results on Web of Science, thus highlighting several geometries of planar antennas that can be used for the implementation of printed LC-based telemetric systems. Furthermore, we identified several methodologies used to print inductors and specific strategies addressing how to improve their performance in terms of transmission quality and *Q*. We discarded several studies specifically focused on particular geometries of inductors used in RFID or NFC systems but not suitable for ordinary passive LC telemetric systems. A second search specifically focused on printed inductors was then performed, thus reporting 17 useful results on Scopus and 15 on Web of Science. Within this search, we excluded all the studies concerning 3D printing of inductors and those addressing power transmission systems that did not present a proper rationale applicable to printing passive LC-based telemetric systems.

Table 1 summarizes the parameters of the conducted search in terms of query on Scopus and Web of Science on 30 October 2025. Table 1 also shows the results for each query and the number of articles taken into consideration to conduct the scoping review. Looking at the results obtained in terms of the number of articles, it is possible to observe that, using queries that can be considered equivalent, more articles were identified in Scopus than Web of Science. Furthermore, all the articles found using Web of Science are present within the search performed on Scopus. Eventually, a total of 34 articles identified through this systematic approach were considered for the scoping review.

It is worth noting that because the included sources were highly heterogeneous and the objective of our scoping review was to map the field rather than compare interventions or derive pooled performance estimates, no meta-analysis was undertaken. Formal risk-of-bias assessment was not performed, consistent with the scoping-review objective. Instead, the evidence was synthesized descriptively and grouped according to materials, manufacturing approach, application area and performance in order to identify recurrent design patterns, methodological trends, and knowledge gaps.

The authors jointly contributed to the organization and analysis of the data from the selected studies. For each of the included studies, certain specific information was extracted using a predefined form, which led to the creation of two tables. More specifically, the first table shows the conductive materials used, the substrates, and the sintering methods used for each technology, whereas the second table is focused on the geometric layouts of the antennas in terms of shapes, number of turns, and covered areas. Furthermore, we also specifically report the ranges of the electrical quantities, in terms of the optimal values of inductance (*L*), resistance (*R*) and quality factor (*Q*), grouped by geometrical layout and according to the characteristic frequencies.

## 4. Results

### 4.1. Principle of Operation of the Telemetric Systems and Measurement Methods

**Figure 5:**
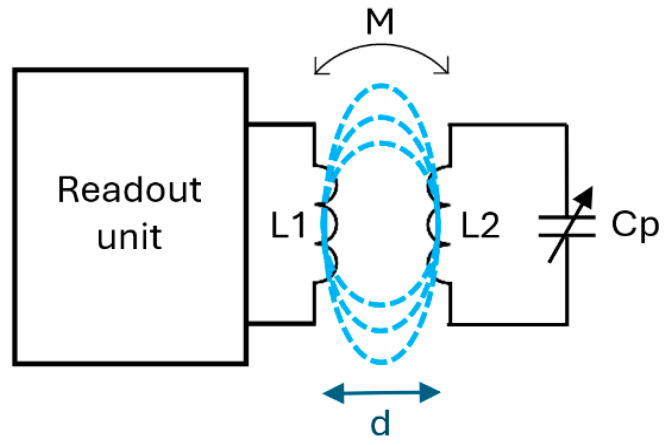
Telemetric system: parallel LC resonant circuit coupled with its devoted readout unit.

The systematic search revealed that the majority of telemetric systems utilize a parallel LC resonant circuit as a passive sensing component. In this configuration, the sensor is magnetically coupled to a readout coil positioned at a fixed distance, *d*. This mutual coupling enables the readout unit to wirelessly power the passive sensor while simultaneously interrogating it. Figure 5 illustrates the schematics of this telemetric system, detailing both the passive LC sensor and the readout interface. The passive LC sensor is composed of *L*_2_ and *C_p_*, whereas the readout unit antenna is composed only of *L*_1_. Focusing on the coupled inductor, *L*_1_ represents the primary coil, whereas *L*_2_ is the secondary coil.

To obtain distinguishable peaks in frequency behaviour, it is necessary to consider the distance *d* between the readout coil *L*_1_ and the secondary coil *L*_2_. It is worth underlining that beyond a certain distance *d*, the magnetic coupling between the secondary *L*_2_ and the readout coil *L*_1_ is insufficient to provide power and perform the measurements. This results in an attenuation of the read signal and, consequently, makes it impossible to correctly identify the peaks in the frequency response. It is therefore necessary to maintain the distance *d* between *L*_1_ and *L*_2_ at an appropriate value so that the peaks in the frequency response can be distinguished and identifiable peaks can be obtained [30,31].

**Figure 6:**
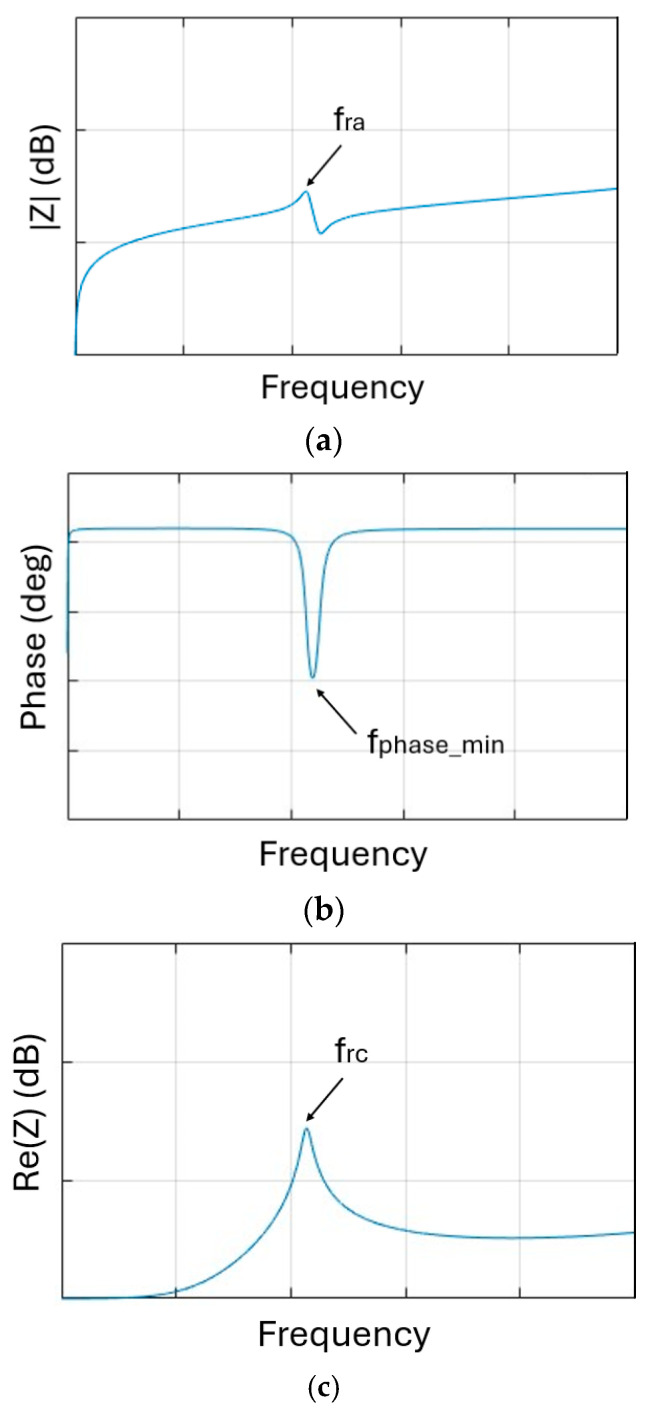
Example of modulus (**a**), phase (**b**) and real part (**c**) of the impedance read at the terminals of the readout unit.

The resonance frequency of the passive LC sensor *f_r_* can be defined as:

(1) $$f_{r}=\frac{1}{2\pi \sqrt{L_{2}C_{p}}}$$

where *L*_2_ and *C_p_* are, respectively, the inductance and the capacitance of the sensor. By measuring the resonance frequency of the LC resonator, it is therefore possible to obtain the quantity of interest measured by the capacitive sensor. The readout unit, exploiting the reading of the impedance at its terminals, must allow the measurement of the resonance frequency of the LC resonator to measure the physical quantity of interest. However, since the coupled inductor composed of *L*_1_ and *L*_2_ is not ideal, it is important to note that the resonance frequency of the impedance read at the terminals of the readout unit is not the resonance frequency *f_r_* indicated in (1). The resonance frequency can also be affected by the environment surrounding the sensor as the dielectric constant of the environment affects the measure of such frequency [12,17]. The resonance frequency read at the terminals of the readout unit will change anyway as *C_p_* varies. The variations in the values of the resonance frequency will therefore be attributable to the variation in the *Cp* values. The resonance frequency at the terminals of the readout unit can be measured in different ways. Two main methods emerge from the bibliographic research. The first method consists of measuring the impedance at the readout coil *L*_1_ coupled to the *L*_2_*C_p_* resonator placed at a fixed distance *d* using an impedance meter as the readout unit. Considering Figure 6, which shows the modulus, phase and real part of the impedance with the resonance frequency *f_ra_*, *f_rc_* and the frequency at minimal phase *f_phase_min_*, in proximity to *f_ra_* and *f_rc_*, the target frequencies are *f_phase_min_* and *f_rc_*. It is possible to identify the frequency *f_phase_min_* as the frequency which minimizes the phase of the impedance [4] in the considered frequency range in Figure 6b. The usage of the impedance meter also leads to the measurement of the real part of the impedance at the readout coil *L*_1_ coupled to the *L*_2_*C_p_* resonator. Using the real part, it is possible to measure the resonance frequency *f_rc_*, shown in Figure 6c, as the one at which the real part is maximum [32] in the considered frequency range.

The usage of telemetric systems is related to the contactless measurement of a physical quantity. As shown in Figure 5, using a capacitive sensor *C_p_*, the variation in the physical quantity is related to the capacitance, which is directly linked to the variations in the frequencies of interest (*f_phase_min_* and *f_rc_*). This leads to the capacitance variation being read. Therefore, by measuring the shift in frequencies seen at the readout coil, it is possible to observe the variations in the sensor, which allow the variations in the quantity measured by the sensor to be observed and quantified.

The second method derives the resonance frequency read from the readout unit from the measurement of the reflection coefficient *S*_11_. This parameter is measured using a vector network analyser (VNA), and it is defined as follows:

(2) $$S_{11}=\frac{Z_{in}-Z_{source}}{Z_{in}+Z_{source}}$$

where *Z_source_* is the impedance of the VNA source and *Z_in_* is the impedance at the readout unit coil seen by the VNA [28]. The resonance frequency is identified as the frequency which minimizes *S*_11_ [28].

### 4.2. Description of the Reviewed Works: Layouts, Construction Technologies, and Materials

This section presents the results obtained from the performed search in terms of inks/pastes as conductive material, substrate, printing and sintering technologies, and geometrical layouts of the printed antennas. Within this framework, Table 2 and Table 3 summarize all the characteristics we considered useful for the realization of both antennas and general telemetric systems by using printed electronics technologies. Then, a presentation of the reported electrical quantities and a description of the fields of application of the systems found in the literature are provided. Table 2, constructed as described in Section 3, shows for each technology the employed conductive materials, the substrates, together with the sintering methods. Indeed, IJP, SP, and AJP represented the main printed electronics technologies to additively manufacture antennas and inductors, with IJP and SP as the most widely used solutions. IJP was specifically reported to allow for rapid prototyping, ensuring the deposition of a thin layer of functional ink with a good throughput only on planar substrates, both rigid and flexible [17,33]. On the other hand, SP allows a huge throughput of thicker printed layers only on 2D substrates [12]. Further, SP allows high-viscosity inks/pastes to be printed, enabling us to obtain printed samples with better electrical properties (e.g., low sheet resistance). However, SP requires the use of stencils, which increases production time and costs [34]. Lastly, AJP has been represented as a printing technology leveraging a higher geometrical resolution with respect to the previous ones. In contrast, AJP is also capable of printing on 3D complex objects and surfaces, as it is possible to print at an adjustable height with respect to the substrate level (1–5 mm) thanks to an airstream-based focusing principle [35]. In addition to the use of AJP alone, it is possible to find applications where AJP has been combined with electroplating [35] and with electrodeposition [36]. Although AJP represents an attractive technology, by using Ag ink the achievable conductivity of thin printed traces is lower with respect to the bulk material [36]. Thus, the combination of electrodeposition or electroplating with the introduction of more conductive materials has been demonstrated to be useful to reduce the equivalent resistance of the traces [35]. Further but less common printing techniques have also been found in our analysis. A piezoelectric Drop-on-Demand IJP was proposed by using a specific printer that instead of filling 2D pixels, as in usual solutions, allowed printing along trajectories specified by the user. The described printing mode was defined as “vector mode” [37]. Furthermore, laser-based technologies are also used to develop printed telemetric systems. For example, Laser-Induced Forward Transfer (LIFT) has been used to print on regular paper [34]. LIFT enables digital printing using laser pulses that transfer ink from a donor substrate to a receiving substrate; using this technique, it is possible to print on flexible or rigid substrates [34]. Another laser-based technology, namely a four-axis self-developed laser sintering machine, has been used to print antennas using silver nanoparticle ink on a polyoxymethylene (POM) substrate [38]. Finally, laser-enhanced direct print additive manufacturing (LE-DPAM) has also been used. The LE-DPAM process combines micro-dispensing to release the conductive ink, FDM deposition to realize the polymeric substrate, and a laser to machine cavities and pattern the element geometry [29].

The printing techniques detailed above represent those explicitly identified by our scoping search for passive LC telemetric systems. However, it is critical to note that several other prominent printing technologies—specifically gravure printing, reverse offset printing, and transfer printing—were absent from the retrieved literature. Despite this, they represent highly promising candidates for the future fabrication of passive LC-based telemetric systems, particularly given their compatibility with the nanoparticle-based silver inks that currently dominate the field. As a high-speed, roll-to-roll (R2R)-compatible process, gravure printing excels in high-volume manufacturing. Its application would be crucial for transitioning printed telemetric tags from lab-scale prototypes to mass-produced, ultra-low-cost commercial smart products. Reverse offset printing, on the other hand, is renowned for its ultra-fine resolution, capable of achieving trace widths and spacings well below 5 micrometres. Utilizing reverse offset printing could allow for the extreme miniaturization of planar inductors while maintaining a high number of turns, thus keeping parasitic capacitance low and the quality factor high. Moreover, the optimal substrate for a high-temperature sintering process is often incompatible with the final smart product. Transfer printing would allow the LC resonator to be printed and cured on a specialized donor substrate before being physically transferred (like a micro-decal) onto highly complex, curved, or thermally sensitive object surfaces where direct printing is unfeasible.

Concerning the materials, the following section presents the findings of the identified research. Silver, both in the form of ink and paste, depending on the printing technology, represents the most common solution. As previously underlined, to enhance the conductivity of AJP-deposited silver inks, Cu electrodeposition and Au electroplating were performed [35,36]. Additional conductive materials we found in our review are represented by multiwalled carbon nanotube ink to perform strain sensing [39] and Al paste [40]. Focusing on the substrates, the most used ones are polyimide (e.g., Kapton) and paper-based solutions. Polyimide was reported to be extremely useful for applications where flexibility of the printed structures is required to specifically conform with the surface, also enabling roll-to-roll configuration [28,30]. Paper-based substrates appeared to be widespread, being biodegradable, ultra-low-cost materials pushing toward a higher overall sustainability of the device [41,42,43]. Other typically used substrates are PEN (polyethylene naphthalate), PET (polyethylene terephthalate), alumina, ABS (acrylonitrile butadiene styrene), glass and bare piezoelectric quartz crystal.

As for sintering technology, traditional thermal sintering, performed in common ovens, was the most common choice. Other identified techniques include photonic sintering, vacuum heating, a hot plate and laser sintering.

The geometric characteristics of the analysed printed inductors are summarized in Table 3, which was compiled as described in Section 3.

The most common geometries include circles, Archimedean spirals, squares, rectangles, and meander dipoles. Non-standard custom geometries can also be considered, such as a combined octagonal and square-shaped coplanar waveguide [44] and a custom circular geometry [45]. The number of turns used to design the inductors ranges from 0.5 to 62. The overall surface for the antennas ranges from a minimum of 340 × 340 μm^2^ [29] to a maximum of 78 × 48 mm^2^ [46].

**Table 2:** Key parameters for inductor production.

Technology	Conductive Material	Substrate	Sintering	References
Inkjet printing (IJP)	Ag-based ink, multiwalled carbon nanotube ink	Paper, glossy photo paper, uncoated paper, polyimide, PEN, photo paper, Kapton, PET, 7 μm thick polyimide spin-coated on 725 μm thick silicon substrates	Hot plate, oven, pulse forge, photonic sintering, vacuum heating, drying, convection air oven, static oven	[17,27,28,30,33,37,39,41,43,44,45,47,48,49]
Screen printing (SP)	Ag-based ink, Ag paste, stretchable Ag paste, Ag micro-flake paste, Al paste	Kapton/polyimide, PET, PI sheet, printer paper, paper, alumina, plastic	Convection air oven, drying, oven, high-temperature energy-saving furnace	[2,12,18,40,42,46,50,51,52,53,54,55]
Aerosol jet printing (AJP)	Ag-based ink, Au-based ink	Bare piezoelectric quartz crystal, alumina	Oven	[19,32,56]
AJP + electrodeposition/electroplating	Ag-based ink, Cu for electrodeposition, Au for electroplating	Glass, alumina	Hot plate	[35,36]
Self-developed laser sintering	Ag-based ink	Polyoxymethylene (POM) covered with a layer of polyimide	Laser sintering	[38]
Laser-enhanced direct print additive manufacturing (LEDPAM)	Ag-based ink	ABS	Laser sintering	[29]
Laser-Induced Forward Transfer (LIFT)	Ag-IJP ink, Ag-SP paste	c-paper, r-paper	-	[34]

**Table 3:** Inductor design parameters.

Technology	Geometry	Number of Turns	Area	References
Inkjet printing (IJP)	Archimedean spiral, square, rectangular, square hexagonal, square octagonal, meander, circular, combined octagonal and square-shaped coplanar waveguide, custom circular geometry	2, 2.5, 3, 4, 5, 5.5, 7, 25, 10, 15, 20, 62, 6	From (8.1/2)^2^ × pi mm^2^ to 61 × 61 mm^2^	[17,27,28,30,33,37,39,41,43,44,45,47,48,49]
Screen printing (SP)	Rectangular, square, Archimedean spiral, spiral	15, 7, 9, 4, 1, 4	From 1 × 1 cm^2^ to 78 × 48 mm^2^	[2,12,18,40,42,46,50,51,52,53,54,55]
Aerosol jet printing (AJP)	Spiral	9, 5, 10	From less than 1 cm^2^ to (6)^2^ × pi mm^2^	[19,32,56]
AJP + electrodeposition/electroplating	Spiral	1, 2, 3, 4, 27	-	[35,36]
Self-developed laser sintering	Archimedean spiral	2	-	[38]
Laser-enhanced direct print additive manufacturing (LEDPAM)	Rectangular	0.5, 1, 2.5, 3	From 340 × 340 μm^2^ to 750 × 750 μm^2^	[29]
Laser-Induced Forward Transfer (LIFT)	Square	6	-	[34]

As previously underlined, it is also fundamental to identify the ranges of the electrical quantities found in the literature. In particular, we hereinafter report the ranges of the optimal values of inductance *L*, resistance *R* and quality factor *Q*, grouped by geometrical layout and according to the characteristic frequencies. Here we start with the inductance values:

- *Circular and spiral geometry*: the most representative values are 3 μH at 5 MHz [32], 229.3 nH at 110 MHz for the 110–190 MHz range, 62.1 nH at 205 MHz for the 205–278 MHz range, and 45.81 nH at 306 MHz for the 306–395 MHz range [35];
- *Archimedean spiral geometry*: an inductance of 337 nH was measured at 162 MHz [17];
- *Square spiral geometry*: in the 10–20 MHz measurement range, the value reported is 11.5 μH at 20 MHz [28];
- *Rectangular spiral geometry*: in the 13.56–50 MHz range, an inductance of 7.88 μH was measured at 13.56 MHz [2].

Then, we report the resistance values of the printed solution, grouped again by geometrical layout:

- *Circular and spiral geometry*: from 1.6 Ω [35] to 61 Ω [36];
- *Square spiral*: from 3.8 Ω [42] to 12.7 kΩ [27], reaching a resistance of 9.73 MΩ in the case of [39];
- *Rectangular spirals*: from 28 Ω [50] to 367.42 Ω [51].

Lastly, quality factor *Q* values are reported as follows, grouped by geometrical layout:

- *Circular and spiral geometry*: from 9.59 [35] to 25.09 [35];
- *Square spirals*: from 1.5 [42] to 9.4 [34];
- *Rectangular spirals*: from 2.08 [46] to 21 [29].

The analysis of the telemetric systems found in the literature shows that most of them use capacitive sensors, while in [39,44] the inductor itself is used as the sensing part, whereas in [54] a commercial sensor was used. Depending on the application and the use of the telemetric sensors, different reading distances have been identified. In particular, the reading distances reported range from 2.5 mm [41] to 2 cm [28,45]. From an application viewpoint, the analysed systems were employed in humidity sensing [2,43,45,46], potassium ion sensing [28,30], fluidic pressure sensing [17], strain sensing [39,41,51], temperature sensing [18,54], gas sensing [40], fluid level sensing [12], pressure sensing [52], SARS-CoV-2 detection [53], liquid/acetone water detection [44], structural health monitoring [48], health monitoring of polymer composites [49], human motion detection [33], and spaceborne applications [56].

## 5. Discussion

### 5.1. Geometrical and Layout Aspects

The inductance values of the inductors are influenced by their geometrical layouts, the number of turns and the spacing between consecutive traces and trace width. In particular, the circular geometry allows higher inductance values to be obtained for the same area occupied by a square or octagonal geometry [37,47]. To predict the inductance value of the inductors obtained, it is possible to use the closed formulas available in the literature for circular, Archimedean, square, square hexagonal, square octagonal and meander planar spiral geometries [33,47]. These formulas consider the width and thickness of the conductive trace and the spacing between the turns of the spirals: the higher the number of turns, the higher the value of the inductance.

Furthermore, the behaviour and values of the inductors can be accurately estimated prior to fabrication by using different simulation tools, such as Keysight Advanced Design System (ADS) [2,29,51,52,55], COMSOL Multiphysics [2,45,55], Ansys HFSS [29,30,50,56], Computer Simulation Technology (CST) Microwave Studio Suite (MWS) [44] and in-house software [34]. The simulations carried out in the analysed works were performed on square [30,34,52], rectangular [2,29,50,51,55], spiral [56], combined octagonal [44] and square-shaped coplanar waveguide and custom circular geometry [45]. Furthermore, the technologies used for the development of the inductors are SP [2,50,51,52,55], IJP [30,44,45], AJP [56], LEDPAM [29] and LIFT [34]. While the reviewed studies generally reported good coherence between simulated and experimental results, a closer assessment of practical implementations and prediction accuracy reveals distinct trends. Typically, predictive models for inductance (*L*) demonstrate high accuracy, as *L* is primarily governed by macroscopic geometric parameters. Conversely, predicting the equivalent series resistance (*R*) and the resulting *Q*-factor can be significantly more challenging. In fact, simulation tools frequently underestimate *R* (and consequently overestimate *Q*) because they default to idealized, smooth trace profiles and uniform bulk conductivities. In practical application, printed traces exhibit variable morphology, edge roughness, ink spreading, and non-uniform nanoparticle sintering. Furthermore, accurately predicting parasitic capacitance (*C_p_*) requires highly precise modelling of the substrate’s specific dielectric losses and the exact physical footprint of the printed traces, which often deviate from the nominal CAD design due to ink wicking. Therefore, while simulations are excellent for initial geometric tuning, researchers must anticipate that experimental *Q*-factors can fall below the idealized values estimated through simulated predictions.

**Figure 7:**
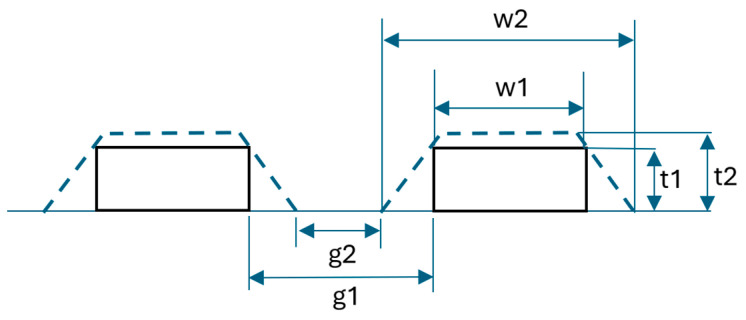
Widening of the conductive trace caused by multiple depositions.

Another important parameter to consider is the quality factor *Q* of an inductor, given as:

(3) $$Q=\frac{\omega L}{R}$$

where *L* and *R* are, respectively, the inductance and the equivalent resistance of the coil, while *ω* is the operating angular frequency [12]. From (3), it can be stated that the quality factor *Q* is strongly influenced by the value of the equivalent resistance of the inductor *R* [38]. Furthermore, since *Q* represents the ratio between the energy stored and the energy released, having a higher value of *Q* is thus mandatory in order to use them in RF applications [35]. Thus it is necessary to ensure that the resistance has values that are as low as possible [35]. One method to reduce the value of the equivalent resistance of the inductor is the enhancement of the conductivity of the traces by increasing the amount of conductive material. The increase in the silver content of the conductive paste implies a decrease in the resistance [12]. Another strategy is to increase the area of the section of the conductive trace, since the value of *R* considering a conductive trace is given by:

(4) $$R=\rho *\frac{l}{S}$$

where *R* is the resistance, *l* is the length of the trace, *S* is the cross-sectional area of the trace (width *w* multiplied by the thickness *t*) and *ρ* is the resistivity of the conductive material. For SP samples, another factor that increases *Q* was related to the use of a larger screen width, since this increases the line width [12]. With IJP, a solution to decrease the equivalent resistance of the inductor was to increase the thickness of the line by introducing multiple-layer deposition, although this method could also lead to an unwanted expansion in linewidth [37]. Theoretically, increasing the thickness of a trace should not result in further geometrical changes. However, as shown in Figure 7, the deposition of several layers could also lead to an uneven enlargement of the printed traces. This variation could further lead to a decrease in the spacing between the turns of the inductor. For this reason, this effect must be considered when inductors within the micrometre range are realized by using IJP techniques. The possibility of increasing the thickness of the line using an increased number of deposition layers in the case indicated above is therefore limited [37].

Even in the case of SP, the use of additional layers was proposed in order to lower the equivalent DC resistance of the antenna [42]. A two-step deposition procedure that combines AJP and electrodeposition of Cu onto printed Ag was proposed, leading to a decrease in resistance by a factor of 35–45× compared to bare AJP depositions [36]. The use of AJP with selective gold electroplating was proposed to reduce the resistance of the printed lines, thus leading to an increase in *Q*. A three- to fivefold improvement in *Q* has been achieved with respect to bare AJP-printed antennas [35].

Beyond the geometric layout of the traces, the physical and electrical properties of the chosen substrate can heavily influence the ultimate performance and reliability of the printed inductor. A systematic analysis of these properties reveals several critical design guidelines. First of all, the surface topology of the substrate fundamentally impacts print resolution. Rough, porous substrates, such as uncoated paper, can cause capillary wicking and deep penetration of the conductive ink, which often requires careful thermal management during printing to prevent short circuits [41,43]. If unmitigated, this leads to uneven trace edges and wider-than-intended lines. This not only limits miniaturization but can also unpredictably increase the parasitic capacitance (*Cp*) between adjacent turns [27]. Conversely, ultra-smooth substrates like glass or polyimide facilitate precise, high-resolution printing with well-defined trace boundaries [28,30], though advanced techniques like aerosol jet printing (AJP) can help mitigate the effects of surface irregularities [35]. Furthermore, the thermal stability of the substrate establishes the maximum allowable sintering temperature for the conductive inks. While polyimide can withstand the high temperatures required for optimal silver nanoparticle sintering, substrates like PET or paper require low-temperature curing, which inherently allows for printing on diverse polymers [51] but results in lower final conductivity [35]. Moreover, during the thermal curing process or operational flexing, mismatches in the coefficient of thermal expansion and mechanical moduli between the metallic ink and the polymeric substrate can induce mechanical stress, risking micro-cracking in the traces and a subsequent degradation in electrical performance [50,51]. Additionally, at the high operating frequencies typical of telemetric systems, the dielectric constant and loss tangent of the substrate play a crucial role. In fact, substrates exhibiting high dielectric losses absorb electromagnetic energy from the near-field, a phenomenon that must be modelled as it directly lowers the system’s overall Q-factor and reading range [29]. Additionally, the resonance frequency is highly susceptible to the dielectric constant of the surrounding environment [12,17]. Because hygroscopic substrates like paper readily absorb environmental moisture, their dielectric constant varies significantly—a property explicitly exploited for humidity sensing [2,43,45,46]. However, in non-humidity applications, this moisture uptake can unintentionally detune the LC resonator by shifting its resonance frequency, confusing the readout unit if multi-parameter environmental compensation strategies are not deployed [6,7].

Overall, the reviewed studies showed that geometrical optimization cannot be reduced to maximizing inductance alone. Increasing the number of turns or reducing the footprint may improve compactness or nominal inductive behaviour, but it also affects conductor length, equivalent resistance, trace spacing, and ultimately the achievable quality factor. Therefore, geometry should be interpreted as part of a coupled design trade-off involving occupied area, resistance, operating frequency, and interrogation robustness. In this sense, no single layout emerges as universally optimal; rather, the most suitable geometry depends on the targeted balance between miniaturization, electrical performance, and manufacturability. Furthermore, the choice of the “perfect match” between inks and substrates remains essential, since their material and surface properties ultimately define the physical boundaries within which these geometrical optimizations can be practically realized.

### 5.2. Integration Issues

**Figure 8:**
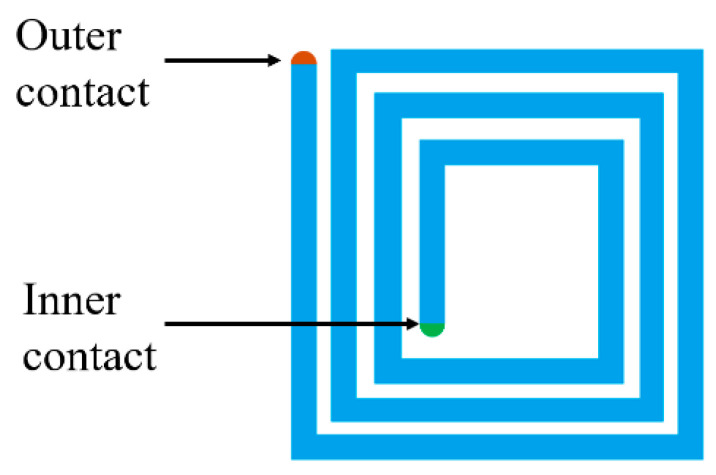
Planar square spiral geometry with highlighted outer and inner contacts.

**Figure 9:**
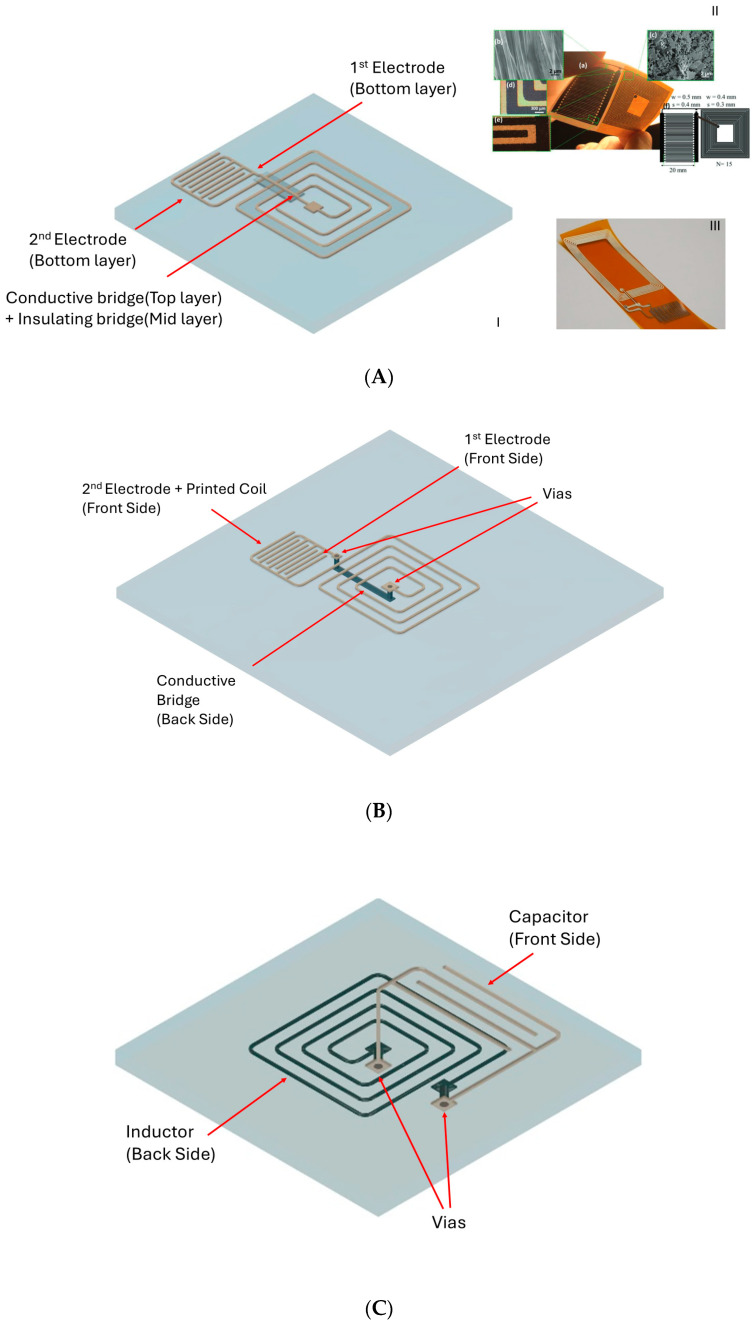
Contacting strategies between coils and sensors: (**A**) Bridging of the inductor internal contact with one pad of a sensor thanks to an insulating layer over the coil. (I): Schematic illustration; (II): visual samples of Figure 2 from [52], published by Advanced Electronic Materials, License CC BY 4.0, 2022; (III): visual samples of Figure 2 from [46], MDPI Sensors, License CC BY 4.0, 2018. (**B**) Use of conductive bridge and vias to make the internal contact of the inductor accessible. (**C**) Double face layout using vias to short-circuit the internal and external contacts of the inductor with a sensor.

In general, the production of printed planar inductors requires vias or bridges to make the terminals of the geometries accessible. Only in this way it is possible to interface the inductor with the sensors to produce a proper passive RL-based telemetric system. Figure 8 shows the outer and inner contacts of a square spiral. Via holes and bridges are therefore necessary to interface the inductors with the sensors (Figure 9).

There are different assembling and bridging connecting methods to connect the internal contact of the inductors to the contact of the sensors. As shown in Figure 9A, the strategy to make bridges involves the use of a conductive printed trace connected between the internal contact of the inductor and the contact of the sensor. It is possible to use a silver conductive trace printed with IJP on polyimide and then glue it using different materials like epoxy adhesive and conductive epoxy resin, followed by a dedicated oven curing [2] or conductive glue [52]. Dielectric insulating materials, mainly polymers, were widely used to protect and insulate traces, avoiding oxidation and giving them toughness and mechanical resistance, as well as allowing multi-layer bridging. The latter enables the possibility of isolating the bridge that connects the inductor contacts by using a dielectric paste [18]. As shown in Figure 9B, another strategy is to connect the internal contact of the inductor to one of the sensor terminals thanks to conductive vias and a printed conductive trace on the backside of the substrate [41]. The vias can be realized by drilling the holes with a laser and filling them with a silver-flake-based screen-printing paste. As shown in Figure 9C, it is also possible to print the sensor on one side of the substrate and the antenna on the other, realizing contact between the two by exploiting the use of vias. This method has been exploited in [43], which presented a space-saving printed system that featured an interdigitated capacitive sensor and an inductor printed with IJP. Vias were made using a laser to drill holes, which were then filled with SP silver paste and finally cured in an oven. Despite being quite widely diffused, the use of bridges and vias in printed technology is still a difficult operation [45]. In particular, the usage of vias leads to increasing complexity of inductor production; thus, strategies to simplify the production process were also carried out. Furthermore, since no analyses have been conducted regarding the mechanical stress resistance and durability of these connection solutions, it would be necessary to conduct specific studies on these issues. Therefore, it is necessary to study new antenna geometries or particular sensor designs. For example, in [45] a new inductor layout was presented and studied so as to test its applicability for an LC telemetric system. In particular, the new inductor layout allowed the production process of the system to be simplified by not requiring the use of vias for the connections of the sensor to the inductor. In order to solve this problem, the coil was composed of circles with a common centre and decreasing size with respect to their relative distance from the centre. The circles were connected alternately from one side to the other by one point. This layout allowed the inductor terminals to be directly accessible without the need for bridge or vias to connect to them [45]. In [17], an LC resonator with a capacitive pressure sensor consisting of a series of two capacitors was presented. This work proposed an effective alternative to the use of a single parallel-plate capacitor, which usually requires the use of vias to connect the inductor to the sensor. In this study, the series of two capacitors was achieved by using a common electrode. The proposed configuration allowed the authors to connect this structure to the inductor without the use of vias. This design presented a simplified production of the LC system compared to standard designs, which usually include a single parallel-plate capacitor. An additional aspect to consider in the production of the inductors is related to the possibility of unwanted changes in the inductance due to variations in the quantities to be measured. To avoid this problem, the use of an insulating layer deposited on the inductor was proposed [40]. In other cases, it was considered whether the used materials could be coated onto the substrates, or if membranes of materials could be applied to perform sensing. In these cases, the inductor could be produced separately from the sensor and then connect them with external wires [45]. Alternatively, the inductor could be produced on the substrate, and subsequently the application of the membrane can be realized [51]. In several cases, the printing process was carried out on both sides of the substrate. In the case of paper-based substrates or, more generally, a material capable of absorbing liquids, it was necessary to keep the substrate at a certain temperature, so as to prevent unwanted short circuits between the traces printed on the two sides of the substrate. For example, in [43], an LC system was realized by printing the inductor and the capacitive sensor on opposite sides of a paper substrate. To prevent the ink from penetrating too deeply into the paper, the substrate temperature was set to 55 °C to allow the solvents to evaporate more quickly. A further study also reported the same method to avoid the problem of ink penetration into the paper substrate [41].

A key outcome of the reviewed literature is that integration is often a greater bottleneck than the resonator design itself. Although bridges, vias, and double-sided layouts enable practical connection between the inductor and the sensing element, they also increase fabrication complexity and may reduce mechanical robustness and reproducibility. This suggests that future improvements should not focus only on the electrical optimization of the inductor but also on architecture-level simplification strategies capable of reducing the number of interconnection steps. In this respect, layouts specifically conceived to avoid vias or minimize multi-layer routing appear particularly promising for the development of robust printed telemetric systems.

### 5.3. Technological and Production Issues

Within this scoping review, several factors were identified as capable of influencing the electrical characteristics of the printed components. Different conductive materials in the form of inks and pastes can be identified, but the most used are silver-based. Silver-based conductors are widely available on the market [36] and are commonly used due to their low sintering temperatures [35,51]. The use of low sintering temperatures makes it possible to print with silver-based conductors on different types of polymeric substrates [51]. However, silver-based conductive inks are mainly composed of nano/microparticles of functional conductive material; these present a lower value of conductivity with respect to bulk conductors [35]. A lower conductivity of the inks leads to an increase in the resistance of printed conductive traces, thus further leading to a lower value of the *Q*-factor. In light of this, devoted strategies to reduce the resistance were proposed, including combining processes, as previously described for AJP with electrodeposition or electroplating [35,36]. However, the use of these combined processes increases production complexity as it requires two sequential steps.

Other alternative strategies can be employed, such as multi-layer IJP, which can reduce equivalent *Rs* values [34].

IJP is a printing method that can achieve higher trace resolutions with lower production complexity. However, despite achieving higher trace resolutions and more windings, multi-layer IJP still results in higher equivalent resistances than can be achieved with SP using the same geometry. An example of this phenomenon is provided by the results reported in [52], which show that a single SP pass achieved the same Q as five IJP layers. In [57], it is also reported that, with the same geometry, inductors with a lower Q-factor were obtained when printing with IJP than with SP. To obtain a reduction in the width of the printed traces, an alternative to the use of IJP is AJP, which also leads to good printing results even in cases of irregularities of the surface of the substrates in use [35]. However, AJP can run into performance issues in terms of equivalent resistance achievable for printed inductors [35,36].

There is also a trade-off to consider between performance improvement and the feasibility of implementing the proposed solutions in an industrial setting. The use of printing technologies such as IJP and AJP, being time-consuming techniques, is certainly not the most suitable approach for industrial implementation. Therefore, the use of IJP to create geometries that utilize multiple layers, or AJP combined with electroplating or electrodeposition, not only introduces greater complexity but also leads to longer production times. A technology better suited for industrial implementation is certainly SP, as it allows for faster printing and facilitates the development of printed inductors with potentially better performance. This study also reveals that the examined studies give little emphasis to long-term stability or environmental influences on the systems. Further research and analysis in this area would therefore be a great opportunity to deepen such aspects.

While silver nanoparticle inks dominate the current literature, their inherent limitations regarding conductivity and required sintering temperatures pose different critical challenges. Consequently, the application of emerging material systems, specifically gallium-based liquid metals (EGaIn) and liquid-metal-particle-based inks, represents a major, largely unexplored frontier for printed passive LC telemetric systems [58,59]. Recent advances have demonstrated that high-resolution, reconfigurable liquid metal printing can achieve near-bulk conductivity, fine feature sizes, and sintering-free processing. For passive LC telemetry, the adoption of liquid metal printing is not only highly possible but directly addresses current technological bottlenecks. The exceptional conductivity would inherently maximize the inductor *Q*-factor and reading range without requiring multi-pass printing. Furthermore, the sintering-free nature of LM processing is perfectly suited for temperature-sensitive flexible substrates. Most promisingly, the ability to print 3D, out-of-plane liquid metal architectures could revolutionize inductor design by creating free-standing conductive arches, completely eliminating the need for complex, failure-prone printed vias or multi-layer dielectric bridges. Validating the long-term stability and magnetic coupling efficiency of LM-based inductors stands as a critical and highly lucrative gap for future research. Indeed, further study of these materials and techniques is therefore necessary to determine their future potential for improving printed passive LC-based telemetric systems in terms of performance and size.

Taken together, these results indicate that the choice of material and printing technology is governed by a multi-parameter trade-off rather than by a single performance criterion. Screen printing appears more suitable when low resistance, thicker deposits, and industrial scalability are prioritized, whereas inkjet printing offers greater design flexibility and prototyping capability at the expense of higher equivalent resistance. Aerosol jet printing is particularly attractive for complex or non-planar substrates, but its electrical performance often requires additional post-treatments, which increase process complexity. As a consequence, the fabrication route should be selected not only according to the targeted electrical performance but also considering substrate compatibility, production throughput, and the final integration scenario.

### 5.4. Main Design Trade-Offs, Current Limitations and Research Gaps

Across the reviewed studies, the design of printed chipless passive inductively coupled LC telemetric systems is consistently governed by three main trade-offs, as previously underlined. The first is between electrical performance and miniaturization, since compact layouts are attractive for smart-product integration but often penalize magnetic coupling and increase sensitivity to fabrication tolerances. The second is between conductivity and process simplicity, because routes capable of improving Q, such as multi-layer deposition or post-print metallization, also increase production complexity. The third is between fabrication flexibility and industrial scalability, as techniques enabling high customization or non-planar printing are not always the most suitable for fast and cost-effective large-scale production.

The literature also highlights several limitations of the presented current approaches. Most studies remain proof-of-concept demonstrations and are validated under highly specific experimental conditions, which makes direct comparison between devices very difficult. In particular, reported values of inductance, resistance, *Q*-factor, and read distance are strongly affected by differences in geometry, operating frequency, material formulation, substrate, and readout configuration. Moreover, limited attention is generally paid to long-term stability, environmental durability, and the mechanical reliability of interconnection strategies such as vias, bridges, conductive adhesives, and multi-layer structures. In fact, there is a specific gap in understanding how environmental factors (such as humidity and temperature variations) and cyclic mechanical stress influence performance over time. Specifically, there is a critical need to systematically investigate the mechanical reliability and durability of interconnected solutions to ensure consistent performance in real-world deployment. In this context, in-mould electronics (IME) can be further exploited for durable smart products, and the exploitation of liquid metals can ensure current conductivity limits are overcome.

Based on the performed analysis, the main research gaps concern: (1) the lack of standardized reporting and benchmarking criteria for printed LC telemetric systems; (2) the need for stronger reader–sensor co-design rather than isolated optimization of the resonant element; (3) the limited study of durability, ageing, and environmental stability under realistic operating conditions; and (4) the need to translate promising laboratory prototypes into more reproducible and scalable manufacturing workflows. Therefore, future research should move beyond demonstrating functional devices and focus more explicitly on integration robustness, comparability of results, and design rules for application-oriented smart-product implementation.

## 6. Conclusions

The usage of PE technologies can help in the diffusion of smart products, thanks to the ease of printing onto well-known substrates (e.g., polyimide and paper), and then integrating them in different contexts. Otherwise, direct printing and implementation onto existing devices may be possible. Given these characteristics, it is possible to implement printed telemetric systems in contexts where it would not be possible to insert wired solutions. In several cases, it is therefore possible to use such systems in contexts where traditional sensors cannot be implemented. A key component, besides the sensor, is the transmitting antenna, which affects the system quality. The highest possible antenna quality factor is needed to improve the overall performances of the telemetric system. Thus, studying antenna design in PE is necessary to improve telemetric system performance. This review allows the implementation aspects of printed telemetric systems to be deepened, with particular attention to the production of printed antennas. It has been highlighted that the most common printing techniques employed in the analysed works are IJP, SP and AJP. Eco-friendly telemetric systems can be produced thanks to the use of biodegradable substrates such as paper, which is also low-cost and flexible, making the systems implementable in different application areas such as food packaging. Other flexible substrates such as polyimide or paper allow the systems to be conformed to various surfaces. On the other hand, in terms of transmission quality, the quality factor *Q* of printed telemetric systems needs improvement strategies due to its poor values. This means, other than designing the geometrical layout of the antenna and selecting the proper functional conductive material, the printing technique must also be managed. Different design strategies emerged. The design of the telemetric system must consider the target substrate on which the printing is to be carried out. In the case of non-planar surfaces, it is mandatory to use AJP. However, this can lead to a low *Q*, so post-treatments have to be taken into account (i.e., electroplating or electrodeposition). In the case of planar substrates, IJP and SP are the most suitable technologies. In the case of IJP, it is possible to obtain a higher resolution, but it is necessary to consider that, unlike SP, even in the case of multi-layer printing, a lower *Q* can be achieved with respect to SP, regardless of the geometry. In general, as far as the production of the printed telemetric system is concerned, it is also necessary to consider how to interface the sensor with the inductor used as a transmission antenna. The connection strategy to connect the inductor to the sensor must be considered during the design phase, using vias or bridges applied between the contacts. The decision will have an impact on the complexity of the production of the telemetric system.

The works exposed in this article will be a starting point to explore the available printing techniques to obtain the best performance in terms of *Q*-factor, inductance values and equivalent resistance values. In particular, it is necessary to identify both layout and printing methods to optimize the values of the electrical quantities of the printed inductors. This decision must be made considering the type of surface on which the system has to be implemented.

While the fundamental building blocks of printed passive LC-based telemetry have been established, transitioning these systems from laboratory prototypes to proper “smart” products requires overcoming several open challenges and exploiting emerging opportunities. A primary future perspective is the development of fully printed passive LC telemetric systems capable of performing multi-parameter measurements within a single, highly miniaturized footprint. Overcoming the cross-talk and mutual inductance issues inherent to dense, fully printed sensor arrays remains a critical open challenge. Moreover, as the IoT expands, electronic waste becomes a pressing concern. Eco-friendly telemetric systems can be produced thanks to the use of biodegradable substrates, such as paper. A major emerging opportunity lies in coupling these substrates with transient, biodegradable conductive inks to create fully compostable telemetric tags for temporary monitoring in agricultural, environmental, or food packaging applications. In this context, while passive LC systems currently operate without onboard power storage by relying on the magnetic field of the readout unit, an exciting frontier is the integration of printed energy-harvesting modules (e.g., printed piezoelectric or triboelectric generators). Such integration could temporarily boost the reading range or power localized active sensing elements before transmitting data passively. Another significant future perspective is the direct integration of printed telemetric systems into structural plastic parts via in-mould electronics. However, deeper knowledge is required regarding the selection of suitable materials. Specifically, research is needed to fully understand how aggressive IME production processes, such as high-temperature thermoforming and high-pressure overmoulding, physically and electrically affect the printed inks and substrates.

## Figures and Tables

**Table 1:** Research parameters and results.

**Scopus Research Parameter**	**Number of Results**	**Valid Results**
TITLE-ABS-KEY (printed AND (RFID OR inductive OR contactless OR battery-free) AND (sensor)) AND PUBYEAR > 2017 AND PUBYEAR < 2026 AND (EXCLUDE (EXACTKEYWORD, “Printed Circuit Boards”))	462	17
TITLE-ABS-KEY (printed AND inductor) AND PUBYEAR > 2017 AND PUBYEAR < 2026 AND (EXCLUDE (EXACTKEYWORD, “Printed Circuit Boards”))	330	17
**Web of Science Research Parameter**	**Number of Results**	**Valid Results**
ALL = (printed AND (RFID OR inductive OR contactless OR battery-free) AND sensor) NOT ALL = (printed AND circuit AND board) AND PY = (2018–2025)	485	15
ALL = (printed AND inductor) NOT ALL = (printed AND circuit AND board) AND PY = (2018–2025)	228	15

## Data Availability

The protocol of the review is registered on Open Science Framework at the following link: https://osf.io/wb36t/overview?view_only=2904e002665c427a8ece69c61e160348 (accessed on 6 April 2026).
